# The impact of patient enrolment in primary care on continuity and quality of care around the world, 2014–2024, and lessons for Australia: a scoping review

**DOI:** 10.5694/mja2.52648

**Published:** 2025-04-15

**Authors:** Shona M Bates, Jialing Lin, Luke Allen, Michael Wright, Michael Kidd

**Affiliations:** ^1^ International Centre for Future Health Systems, UNSW Sydney Sydney NSW; ^2^ UNSW Sydney Sydney NSW; ^3^ University of Oxford Oxford United Kingdom

**Keywords:** Primary care, General practice, Health policy, Health systems, Practice management, Primary health care, Continuity of patient care

## Abstract

**Objectives:**

To identify publications examining the enablers of and barriers to patient enrolment in primary care and its impact on continuity and quality of care; to assess the likely effectiveness of voluntary patient enrolment (MyMedicare) in Australia with regard to improving continuity of care and supporting other health care reforms.

**Study design:**

Scoping review of peer‐reviewed journal article published in English during 1 January 2014 – 12 July 2024 that evaluated primary care enrolment models, including patient enrolment enablers and barriers.

**Data sources:**

PubMed, Cochrane Database of Systematic Reviews, Embase, CINAHL (Cumulated Index in Nursing and Allied Health Literature), PsycINFO, PAIS (Public Affairs Information Service), Web of Science, Scopus. The bibliographies of included articles were checked for further relevant publications.

**Data synthesis:**

The database searches and bibliography checks identified 508 potentially relevant articles; we reviewed the full text of 66 articles after title and abstract screening, of which 24 publications met our inclusion criteria. Twenty‐two of the included studies were undertaken in fifteen countries, including eleven in Canada, four in Australia, and two each in the United Kingdom and New Zealand; one publication compared schemes in twelve countries, one was a rapid review. The characteristics of patient enrolment models differ greatly between countries in both form and implementation, including the mandatory and voluntary components. We found little evidence that enrolment improved continuity of care. However, existing patient engagement with usual general practitioners was high among participants in many studies, and some studies involved patients who may already have had high levels of continuity of care. There is evidence that enrolment can support primary care reforms, including preventive care and the management of chronic conditions, and that other reforms, such as incentives and increased access to services can affect the enrolment of patients and practices. People in marginalised groups or with complex care needs are less likely to enrol with practices or practitioners.

**Conclusions:**

The Australian voluntary patient enrolment scheme should be continuously evaluated to assess levels of engagement by patients and general practices, drawing on the experiences of other countries in which similar schemes operate. Further assessment of overseas enrolment systems could identify reasons for the different experiences reported, as well as enablers of and barriers to successful implementation and better health outcomes.

Primary care services in Australia and overseas are challenged by population growth, ageing populations, inadequate financial support, and workforce shortages; these challenges in turn place pressure on other, more expensive components of health care, including emergency departments and hospitals.^1^ Many governments are responding by seeking greater equity, efficiency, and effectiveness in health care delivery by reforming both preventive and responsive primary care.^2^ In Australia, this is being achieved by implementing the recommendations of the Strengthening Medicare Taskforce.^3^

Enrolment — linking or registering a person with a specific general practitioner or family physician or with a single general practice — is one component of high performing primary care that benefits patients, general practitioners, general practices, and the community by supporting greater continuity and coordination of care,^4^, ^5^ leading to better health outcomes.^6^ Enrolment provides relational, informational, and management continuity^7^ at a single point of care or medical home.^4^, ^5^, ^8^, ^9^, ^10^ In turn, continuity of care is expected to lead to improved health, reduce inappropriate health service use and costs, and improve patient satisfaction.^5^, ^6^, ^7^, ^10^, ^11^, ^12^, ^13^

Patient enrolment also benefits practices by informing resource allocation,^8^ supporting screening for and managing chronic conditions,^4^, ^8^ and increasing productivity.^14^ Patient enrolment can provide additional information to government agencies that supports health system planning, preventive care, and the development of primary care reforms, including context‐specific funding reforms.^4^, ^8^, ^15^, ^16^, ^17^

Prior to 2023, Australia was among the few Organisation for Economic Cooperation and Development countries to not have a system of patient enrolment.^1^ In 2023, 76.9% of Australians reported they had a usual general practitioner,^18^ but the freedom to seek care from multiple practices is likely to reduce continuity of care, particularly given the lack of informational continuity across practices.^19^ Voluntary patient registration (MyMedicare; Supporting Information, part 1) was introduced in Australia in October 2023 to support continuity of care and provide a platform for primary care funding reform.^20^ MyMedicare is supported by additional Medicare incentives for enrolled patients.^21^

We undertook a scoping review of publications about the enablers of and barriers to voluntary patient enrolment in general practices, and its impact on quality of care, in order to assess the likely effectiveness of MyMedicare with respect to improving continuity of care and supporting other primary care reforms in Australia.

## Methods

A scoping review seeks to establish what is known about the evidence for an intervention or about a research question or concept. We report our review according to the Preferred Reporting Items for Systematic Reviews and Meta‐Analyses Extension for Scoping Reviews (PRISMA‐ScR) statement.^22^

### Study design

We included in our review peer‐reviewed journal articles published in English during 1 January 2014 – 12 July 2024 that reviewed or evaluated primary care patient enrolment. We selected this period to focus attention on recent primary care that may be relevant to reforms in Australia. We included original research articles that reported the form of enrolment and the enablers of or barriers to enrolment; we did not include studies of informal registration, payment models, or registration outside primary care unless they were directly related to patient enrolment (Supporting Information, table 1).

We used a standardised search protocol to identify relevant studies in PubMed, the Cochrane Register of Systematic Reviews, Embase, CINAHL (Cumulated Index in Nursing and Allied Health Literature), PsycINFO, PAIS (Public Affairs Information Service), Web of Science, and Scopus: {‘primary care’ OR ‘general practice’ OR ‘primary health care’ OR ‘primary healthcare’} AND {‘patient registration’ OR ‘patient enrolment’ OR ‘patient empanelment’ OR ‘patient rostering’} in {Title Abstract Keyword}. Searches were conducted by author SB on 12 July 2024.

All search results were entered into an Excel (Microsoft) spreadsheet and duplicate records were removed. The first two authors reviewed the titles and abstracts and removed articles that did not meet the inclusion criteria; articles were included for full text review if the two authors disagreed about their relevance. The bibliographies of included articles were checked for further relevant publications.

### Data extraction and management

The data extraction template included the author, title, publication details, and abstract; for articles considered potentially relevant based on their title and abstract, we recorded the jurisdiction, study objective, method, findings, whether enrolment was voluntary or compulsory, form of enrolment, and any associated reforms and enablers of enrolment. Authors SB and JL reviewed the full text and documented the reasons for excluding articles. This detailed record keeping allowed the analysis to be checked by the co‐authors.

### Data synthesis

The results were analysed thematically and grouped by research question and emerging themes.

## Results

The database searches and bibliography checks identified 508 potentially relevant articles. After removing duplicates and screening their titles and abstracts, we reviewed the full text of 66 articles; 24 met the inclusion criteria for our scoping review (Box 1, Box 2).^23^, ^24^, ^25^, ^26^, ^27^, ^28^, ^29^, ^30^, ^31^, ^32^, ^33^, ^34^, ^35^, ^36^, ^37^, ^38^, ^39^, ^40^, ^41^, ^42^, ^43^, ^44^, ^45^, ^46^ Twenty‐two of the included studies were undertaken in fifteen countries: eleven in Canada, four in Australia, two each in the United Kingdom and New Zealand, and one each in Ireland, the United States, and France. One publication compared schemes in twelve countries (Denmark, France, Germany, Ireland, Israel, Italy, the Netherlands, Norway, Canada [Ontario], Sweden, Switzerland, United Kingdom), and one was a rapid review. The four Australian articles^38^, ^40^, ^45^, ^46^ described trial interventions; they were included because of the similarities of the interventions with MyMedicare. Several schemes operate concurrently in Canada^42^ and Ireland,^23^ facilitating a degree of practitioner and consumer choice, and model comparison.

Box 1 **Identification and selection of articles for inclusion in the scoping review of published studies of the enrolment of patients in primary care, 2014–24**

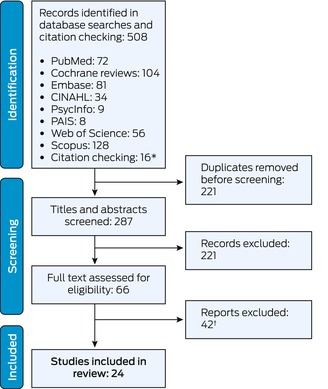

CINAHL = Cumulated Index in Nursing and Allied Health Literature; PAIS = Public Affairs Information Service.

* Of the sixteen publications identified by citation checking, two were excluded after screening their titles and abstracts, and ten were excluded after full text review.

† Detailed reasons for exclusion are provided in the Supporting Information, part 2.

Box 2 **Summary of articles included in the scoping review of published studies of patient enrolment in primary care, 2014–24**

**Table:** 

Reference, location	Study summary
Carmody and Whitford (2007),^23^ Ireland	Overview: Sought views of unregistered patients about registration. Method: Telephone survey of 400 patients about their use of primary care, whether they saw the same general practitioner, whether they saw continuity of care as important, and the characteristics of those who saw other general practitioners. Findings: Patients preferred seeing the same general practitioner or attending the same practice; 89% had regular general practitioners (mean time, 15.6 years), 96% preferred care to be provided in same practice, 16% had consulted general practitioners outside the practice during the preceding year. Larger proportions of young people (18–34 years, 22.7%; 35–54 years, 15.4%; 55–69 years, 9.4%), women (18.5%; *v* men, 9.7%), people who commuted 2 km or more to work (17.8%; *v* less than 2 km, 11%), and of people with poorer health (30.8%; *v* good health, 13.9%) or long term illness or disability (20.8%; *v* no long term illness or disability, 14.4%) had seen other general practitioners during the preceding twelve months. Overall, 81% thought it was important to be registered with a preferred general practice. Consulting general practitioners other than regular practitioner was occasional rather than regular pattern of behaviour determined by circumstances. Voluntary/involuntary: mixed. Form of registration: At time of the study, about 70% of people in Ireland were private patients and were not required to be registered or to visit only one practice; 30% (means‐tested, or under or over age thresholds), who receive free primary care through General Medical Services, were required to be registered. Registration supports planning and funding of General Medical Services. Enablers: Personal preference facilitates continuity of care for most people. Preference based on proximity to work or home and recommendation from family or friend. Respondents indicated they would consider someone other than their regular general practitioner after four visits. Authors suggest patients could opt to be seen as registered patients or not (registration offering additional benefits: invitations to screening).
Dourgnon et al. (2007),^24^* France	Overview: Examined impact of introducing a “preferred doctor” scheme. Method: Survey of patients enrolled in the preferred doctor scheme shortly after introduction of scheme. Findings: 82% of respondents thought it was compulsory, 44% did not want to lose fee reimbursements, 31% said it was to help the government save money, 22% thought it would improve their medical care, and 13% were recommended to register by their doctor. However, 93% of those surveyed already had preferred doctors; 22% who did not declare a preferred doctor attributed non‐enrolment to lack of time or no reason to go to a general practitioner, preference for free choice of physician, or not receiving information. Registration with a particular doctor was not perceived as having any effect. Voluntary. Form of registration: Voluntary preferred doctor scheme alongside existing registration with practices. Enablers: Patients incur additional expenses if not registered with a preferred doctor.
Glazier et al. (2009),^25^ Canada (Ontario)	Overview: Examined measures of access to primary care in different funding models. Method: Examined attachment to a primary care provider (registration) and timely access. Findings: While attachment increased from 91% to 95%, speed of access declined, and emergency department presentations did not change. Difficult to change pre‐existing patterns of use. Biggest impact seems to have been on primary care workforce: income increased, as did the use of multidisciplinary teams. Voluntary/involuntary: mixed. Form of registration: Compared blended capitation and enhanced fee‐for‐service models. Both involve patient rostering, incentives for preventive care, and after‐hours care. Capitation is through age‐ and sex‐adjusted capitation payments, and patient enrolment is mandatory. Enrolment in enhanced fee‐for‐service model is voluntary. Enablers: Compulsory empanelment in capitation model. Higher socio‐economic status associated with enrolment.
Tiagi et al. (2014),^26^ Canada (Ontario)	Overview: Investigated link between patient rostering and emergency department use for non‐urgent care. Method: Analysis of administrative data (patient level, difference‐in‐difference) using control group based on propensity score matching. Findings: Rostering was associated with a statistically significant reduction in non‐urgent visits to emergency department (3% reduction; $8 million saved). The authors also highlighted other elements of the models, such as increased after‐hours services. Voluntary. Form of registration: Three payment models with different incentives and disincentives and services for patients (including after‐hours services). Enablers: Not reported.
Breton et al. (2015),^27^ Canada (Quebec)	Overview: Examined impact of incentives for enrolling patients from a central waiting list, with higher payments to practices for patients with more complex needs. Method: Longitudinal quantitative analysis (five years) of all patients enrolled from central waiting lists. Findings: Physicians favoured enrolment of healthier people over those with greater health care needs and higher assessed priority. Indicates an unintended consequence of registration, and that a different approach may be required to ensure that people with complex needs are enrolled. Voluntary. Form of registration: Family medicine groups (FMGs) and network clinics introduced in early 2000s. FMGs are groups of physicians working with nurses to provide services to registered patients. Government pays for nurses and administrative staff and computer equipment. In 2014, 250 FMGs had enrolled 40% of Quebec residents. Enrolment with other physicians (not FMGs) also introduced. Annual payment of $10 per standard patient, $55 per patient with greater needs (and a per‐visit surcharge). Additional incentive: $103 to register patient with greater needs from waiting list (paid on first and second visits); later increased to $208 for patients with greater needs, and from $0 to $100 for other patients. Enablers: Payments to register patients, higher for patients with greater needs.
Kiran et al. (2015),^28^ Canada (Ontario)	Overview: Evaluation of a shift to blended capitation models and team‐based care on the management of chronic disease. Method: Analysis of population‐based administrative data to compare monitoring and screening of patients enrolled in different models of primary care. Findings: Shift to capitation payments and addition of team‐based care improved diabetes care, but effects on cancer screening were less clear. Voluntary. Form of registration: Various. Enablers: Not reported.
Barker et al. (2016),^29^* United Kingdom	Overview: Examined whether continuity of care improved when patients were offered registration with an accountable general practitioner (in addition to enrolment with the practice). In 2014, this was introduced for patients aged 75 years or older, and from 2015–2016 for everyone. Method: Compared outcomes for patients registered with accountable general practitioners with those for other patients: frequency of visits, tests, and referrals. Findings: 80% of people aged 75–85 years were assigned to named accountable general practitioners; no change in general practitioner contacts or continuity of care, or in tests and referrals, compared with control group. However, whether patients saw their accountable general practitioners could not be determined. Voluntary. Form of registration: General practices were obliged to make reasonable efforts to accommodate patients’ choice of general practitioner. Patients had no obligation to see their named general practitioner. Enablers: Patients are already registered; this shifts registration from practice to an accountable general practitioner.
Christiansen et al. (2016),^30^ United States (northern California)	Overview: Impact of patient empanelment trial (which included team‐based care) on continuity, quality, and efficiency of care. Method: Trial of patient empanelment in federally qualified community health centres (three rural areas, practices serving low socio‐economic status people: Patient Centered Medical Home project); outcomes assessed at six and twelve months. Findings: Empanelment led to 63% of patients seeing their allocated providers. Quality indicators (five health indicators) improved by 9%, and practitioner cycle time by 12 minutes per patient, allowing doctors to see more patients and generate more income. Form of registration: Compulsory empanelment in the trial group. Enablers: Associated with other practice reforms to improve organisation (no incentives, but greater efficiency).
Strumpf et al. (2017),^31^ Canada (Quebec)	Overview: The effect of fee‐for‐service health care costs on the use of family medicine groups (FMGs), which include registration, extended hours, multidisciplinary teams. Examines use of other services, including hospitals, and cost savings. Method: Longitudinal insurance claims database analysis; comparison of patients in FMGs and traditional practices. Characteristics of physicians who work in FMGs; characteristics of patients who do or do not register with FMGs. Findings: Primary care visits declined by 11% per patient per year, specialist visits by 6%, but no effect on hospitalisations or costs of emergency department visits. Registration led to less primary care use but no change in emergency department use. The model is not cost‐effective. The authors also cite an auditor report that the model had not led to changes to clinical practice, organisation, or accessibility of care. Voluntary. Form of registration: Practices decide whether to become FMGs. Enablers: No change in payment mechanisms or sizeable incentives. $7 per patient per year to register older and chronically ill people. General practitioners received $2000–3500 in FMGs, $2000–5500 in non‐FMGs.
Burch et al. (2018),^32^ England	Overview: Examined over‐registration (registered at more than one general practice) and under‐registration (no registration) in primary care by comparing enrolments with census‐derived resident data. Method: Cross‐sectional study comparing population (census) and general practice populations (practice registration); investigated relationship between levels of registration and area deprivation, urbanicity, ethnic background, age, sex, and mean distance to practice. Findings: In England, 3.9% of people were over‐registered (more than two million people), 6% were over‐registered in London (500 000 people). The over‐registration proportion was larger for non‐white residents, women, older people, and people living in areas of higher levels of social deprivation; 67% of recent migrants were probably not registered. As homeless people and recent migrants may not be able to register, the proportion who are under‐registered would be expected to be larger. Mandatory. Form of registration: Patients need to register, but choose where. Patients need general practitioner to access services other than emergency, infectious disease, and some psychiatry services. Enablers: Requirement to register to receive care. Over‐registration associated with groups with high mobility or high health needs. Over‐ and under‐registration may lead to misallocation of resources.
Batista et al. (2019),^33^ Canada (Ontario)	Overview: Examined differences in patient enrolments for recent immigrants for two funding models during 2003–2012. Method: Population pre–post (1985–2003 and 2003–2012) study. Multivariate analysis of registration by immigrant status, adjusted for age, gender, income level, area, and morbidity. Findings: The enrolment rate for immigrants is lower than the overall population rate; the enrolment rate for immigrants is three times higher for capitation‐based models than more advanced family health teams. Women, people in rural areas, and people with few medical conditions are more likely to enrol. Voluntary. Form of registration: In both models, patient enrolment is encouraged. In some capitation practices, physicians can see patients on fee‐for‐service basis (suggested when use of service is high). Enablers: Reformed practices were required to enrol or were strongly encouraged to offer enrolment to their patients.
Singh et al. (2019)^34^ Canada (Ontario)	Overview: Impact of patient rostering and enhanced fee‐for‐service model on care continuity and emergency department presentations. Method: Analysis of longitudinal population‐based administrative data. Findings: Transition to an enhanced fee‐for‐service model led to slight reduction in continuity and coordination of specialised care, probably because physicians worked in groups and shared patients; greater group‐level continuity after the transition. Including an after‐hours component did not reduce non‐urgent emergency department use, possibly reflecting small impact that primary care access has on such visits. Voluntary. Form of registration: Voluntary, fee‐for‐service model. Physicians must provide after‐hours care for rostered patients and work in groups of three or more. Enablers: $5 per patient rostered (existing patients) and $110–180 to enrol new patients (orphaned patients). A small monthly fee per rostered patient and a 10% increase in fee‐for‐service payments. After‐hours care provided.
Breton et al. (2021),^35^ Canada	Overview: Centralised waiting lists (as used in specialist care) to allocate patients to general practitioner enrolment. Seven provinces introduced centralised waiting lists for unattached patients. Method: Logic analysis; step 3 of a larger project, comparing qualitative case study data (step 1) with theoretical aspects (step 2) to identify considerations for design. Findings related to context, intervention, mechanism, or outcome. Findings: Demand for enrolment higher than supply (willingness/capacity of general practitioners to enrol patients). Three waiting list components: patient registration, prioritisation, assignment to a provider. Patients are responsible for registering; prioritisation considers a broad range of conditions and characteristics, and long term acceptability of attachment is important. Identified barriers to attachment: supply of general practitioners, finding general practitioners for patients with complex needs, and reaching unattached patients. Voluntary. Form of registration: Formal registration (Quebec and Ontario have signed agreement in an administrative database) or informal registration (understanding that provider is now the regular source of care). Supported by different payment models. Enablers: Providers must be willing to be engaged; this can be in a process, or having patients registered. Important to engage general practitioners and practices in design to increase its acceptance. Attachment facilitated by care connectors. Barriers include remuneration (fear of false incentives to cherry‐pick), capacity to take on new patients or patients with complex needs, way practice was organised/funded, government/practice relations, and trust in the list system. Concerns about inconsistencies between policy priorities and funding models.
Irurzun‐Lopez et al. (2021),^36^ New Zealand	Overview: Identifying who is not enrolled with a primary care provider. Method: Analysis of administrative data for patients registered and national census data, disaggregated by ethnic background (three categories), age, socio‐economic deprivation, and location. Findings: About 6% of the population is not enrolled (2019); lower enrolment of Māori and people aged 15–24 years, highest enrolment in most affluent areas. Voluntary. Form of registration: Register with single primary health organisation; person deregistered if they do not attend the practice for three years. Enablers: Lower fees when attending registered practice (out‐of‐pocket expenses remain).
Marchildon et al. (2021),^37^ Denmark (DK), France (FR), Germany DE), Ireland (IE), Israel (IL), Italy (IT), the Netherlands (NL), Norway (NO), Canada (CA; Ontario), Sweden (SE), Switzerland (CH), United Kingdom (UK)	Overview: Comparison of registration practices for twelve countries. Described how patient registration was established and evolved, the requirements and benefits for patients, providers, and payers, and its connection to primary care reforms. Method: Desktop review in each country using a common framework to analyse characteristics of patient registration and identify registration and reform problems. Findings: Registration was never introduced in isolation; it was part of broader reforms to improve quality through coordination and efficiency (reducing unnecessary referrals). Limited evidence for the effectiveness of registration. Mandatory registration in three countries. High registration rate achieved by incentives for patients (access to health care, reduced costs, free services) and physicians (capitation payments). Registration means different things in different countries. History and characteristics of the registration system, incentives for patients and providers, and potential for wider use of patient–provider agreements could achieve more timely, appropriate, continuous, and integrated care. Policy makers need to consider local context when introducing reforms based on experiences elsewhere. Voluntary/involuntary: mixed. Form of registration: Mandatory: IT (no choice, initially allocated), IE (medical card holders), IL (members of one fund); mandatory/voluntary (decide where to register or can use private services): UK (general practitioner care only if registered), NL (non‐registered people can be refused appointments, and secondary care access is more difficult), DK, FR, NO, SE (lower fees if registered), CH (lower insurance premiums), DE (some sickness funds provide bonuses). All jurisdictions offered choice and ability to move registration, although some limited by geographic area. Reported association with remuneration based on capitation, or other incentives to provide additional services for registered patients with complex needs. Capitation payments range from 6% in FR to 90% in the UK and 100% in some SE regions. Other forms include fee‐for‐service and pay‐for‐performance. Enablers: Seven countries: part of macro‐level reforms before 1980 (DK, IE, IT, NL, SE, UK). Other countries: part of meso‐level (providing universal health coverage: CA, CH, DE, IL, NO) or micro‐level reforms (improving system integration and care coordination: FR) from the 1990s onwards.
Bonney et al. (2022),^38^ Australia (New South Wales, Victoria, Tasmania)	Overview: Trial of a patient registration and funding model for people with greater health risks (18–65‐year‐old people with chronic illness; people over 65 years of age). Examined patient experiences, particularly relational continuity. Method: Cluster randomised controlled trial (clustered by practice), August 2018 – July 2019. Findings: Patient continuity was high at baseline and not influenced by the intervention. Authors recommend targeting people with specific health risks and low baseline engagement with primary care. Voluntary. Form of registration: Participating patients in intervention practices offered enrolment with preferred general practitioner, a minimum of three longer appointments (capped at $250 per patient), and review within seven days of hospital admission or emergency department attendance. Practices received incentives for longer consultations (dependent on reducing unnecessary prescriptions and tests), early post‐hospital follow‐up (paid on a sliding scale by proportion of patients seen within a week of discharge), and hospital admission reductions (paid on a sliding scale; up to 40% reduction). Enablers: Provided longer appointments and reviews within seven days of hospitalisation.
Lavergne et al. (2022),^39^ Canada (British Columbia, Quebec)	Overview: Examined characteristics of patients aged 40 years or older enrolled in voluntary programs in fee‐for‐service systems in British Columbia and Quebec. Method: Analysis of administrative data for enrolled/not enrolled people. Two programs in Quebec assessed (enrolment of patients with qualifying conditions; enrolment of patients from general population) and three in British Columbia (enrolment of patients with chronic disease; complex care; general population). Examined association between enrolment and neighbourhood income, rural/urban residence, previous treatment for mental illness, previous treatment for substance abuse, and health care service use before program. Findings: For general population programs, likelihood of enrolment was greater in higher income neighbourhoods, but were similar for programs with health‐related eligibility criteria. People with substance use disorders were less likely to register. Enrolled people had more visits and greater continuity of care prior to the program. Voluntary. Form of registration: Various. Enablers: In Quebec, annual payments to practices for registered patients. In British Columbia, additional fee codes and suite of payment incentives for practices that agree to provide longitudinal care to a panel of patients.
Reed et al. (2022),^40^* Australia (South Australia)	Overview: General practitioner intervention for people at risk of poor health outcomes (under 18, 18–64, 65 or more years old). Method: Randomised control trial (randomised by practice). Examined self‐rated health measures and continuity of care with the usual provider of care. Findings: Intervention had no significant effect on the primary outcome (self‐rated health) or secondary outcomes (health literacy, health service use). Economic evaluation found quality of life gain for the two adult groups, but it was not cost‐effective. Authors state it was unclear what would make this approach successful and cost‐effective for practices and patients; they suggested examining other outcomes over longer periods. Voluntary. Form of registration: Enrolment with preferred general practitioner. Enablers: Practices $1000 per patient. Patients: preferred general practitioner, longer appointments, follow‐up within seven days of emergency department presentation or hospital care.
Smithman et al. (2022),^41^ Canada (Quebec)	Overview: When registration introduced, many people could not find doctors accepting new patients. Quebec introduced a waiting list system (2022: 900 000 Quebecois, 10% of population). Evaluated changes in access to and continuity of primary care associated with attachment. Method: Registration and Concentration of Care Index for relational continuity of care at general practitioner and practice level. Findings: After registration, visits to the same general practitioner increased. Overall number of visits also increased; attributed to requirement for initial appointment for registration. Voluntary. Form of registration: Family medicine groups. Enablers: Physicians received registration payment of $19 to $300, according to patient's medical needs.
Bayoumi et al. (2023),^42^ Canada (Ontario)	Overview: Examined association between financial incentives and enrolment of adults with serious mental illness in different enrolment and practice models. Method: Two‐year retrospective cohort study (2016–17 to 2017–18). Compared association between financial incentives and enrolment for adults with mental illness (schizophrenia or bipolar disorder), diabetes, and the general population. Findings: Rostering of patients with significant mental illness was lower than for the general population. Authors suggest that premium payment was not associated with enrolment; there was no incentive to enrol more than ten patients with significant mental illness. Modified capitation payments were based on age and sex, not case mix, thereby providing a disincentive for enrolling people with complex needs. Voluntary. Form of registration: Residents who were rostered or virtually rostered to primary care physicians practising in patient enrolment models. Voluntary, but implemented in different primary care enrolment models. Enhanced fee‐for‐service model (with some bonuses for preventive care), and blended capitation models with and without team‐based care. Enablers: Government premiums for enrolling patients with significant mental illness: annual payment of $1000 in total for the first five enrolled patients and $1000 in total for an additional five or more patients, with the total payment capped at $2000. $12.75 million was paid during study period.
Nabieva et al. (2023)^43^*	Overview: Rapid review of approaches for increasing access to continuous care for patients with no primary care provider. Method: Rapid review. Findings: Identified five distinct themes: financial incentives for patients and providers; health care organisation; policy intervention virtual care and health information technology; medical education. Approaches that increased continuous care had combined two or more of these approaches and reflected the patient medical home model. Form of registration: Not reported. Enablers: Not reported.
Pledger et al. (2023),^44^ New Zealand	Overview: Examined change in enrolment rates in New Zealand during 2016–2023, by sex, age, ethnic background, and socio‐economic deprivation. Method: Quantitative study; analysis of administrative data. Findings: Enrolment increased from 93.5% in 2016 to 95.4% in 2023. Enrolment rates differed by demographic characteristics (lower for Māori, young people, and people from lower socio‐economic status areas). The enrolment of young people increased over time, especially during the COVID‐19 pandemic, but the enrolment rates for Māori, Pasifika, and people from areas of lowest socio‐economic status declined. Enrolment rates increased for wealthy and other ethnic groups (not Māori or Pasifika). Enrolment rates declined during the COVID‐19 pandemic (from July 2019 to July 2023). High enrolment rates do not necessarily mean high levels of preventive care. Vaccination rates (at 24 months of age, used as an indicator of system performance) for Māori and Pasifika also declined over the same period. Voluntary. Form of registration: Enrolment with a single primary health organisation. Enrolment expires if person does not visit for three years. Registration at a second practice cancels prior enrolment. Temporary residents were ineligible for enrolment, as were prisoners disenrolled while incarcerated. Enablers: Enrolment with a primary health organisation provides lower consultation fees and greater continuity of care for the patient.
Javanparast et al. (2024),^45^ Australia (South Australia)	Overview: Staff and patient experiences of a clustered randomised controlled trial of a general practice intervention comprising patient enrolment, longer appointments, and timely follow‐up after hospital care. Method: Qualitative study: 41 practice staff (control and intervention groups); 45 patients from trial sites who had recently been hospitalised. Findings: Mixed views about whether intervention had improved services. Positive changes related to proactive and systematic approach and team‐based care. Patients reported after‐hours care and cost were key reasons to visit emergency department. Post‐hospital follow‐up difficult because of limited communication by hospital. Scheme would not be feasible without additional funding. Trial. Form of registration: Trial only; similar to MyMedicare. Support for appointments with regular general practitioner, access to team‐based care (practice nurse), longer appointments, and more proactive follow‐up after hospital care. Implementation of model supported by additional funding. Enablers: Practice engagement; adequate support funding. Financial barriers hinder practice change.
Reed et al. (2024),^46^ Australia (South Australia)	Overview: Follow‐up at 24 months of one‐year (2018–19) clustered randomised controlled trial of patient enrolment in South Australia. Examined whether there was a time lag in impact. Method: Outcomes assessed were hospital use, specialist service use, pharmaceutical dispensing. Economic evaluation estimated cost per quality‐adjusted life‐year (QALY) saved. Findings: No statistically significant intervention effects for health service use. At twelve months: fewer emergency department presentations, but more hospital admissions and overnight stays (both compared with baseline and with the control group). Estimated incremental cost‐effectiveness ratio overall was $18 211 per QALY gained (lower than $50 000 per QALY gained threshold for cost‐effectiveness); not cost‐effective for all adults (higher costs, lower QALY gain), but cost‐effective for people over 65 years of age (lower hospital costs). Trial. Form of registration: Original trial recruited 1044 people (58 children, 315 adults aged 18–65 years, 671 adults aged 65 years older), identified by their general practitioners as being at high risk of poor health outcomes. Ten intervention, ten control group practices; intervention comprised patient enrolment with preferred general practitioner, longer appointments, timely follow‐up after hospital care. Enablers: Practices received $1000 per enrolled patient for the 12‐month intervention, and $10 000 for administrative costs and patient recruitment and participation in the trial.

* Publications identified by checking bibliographies of eligible publications identified by database searching.

### Characteristics of patient enrolment schemes

Patient enrolment was not introduced as a standalone intervention in any country. It was introduced as part of macro‐level reforms that provided universal health care coverage before the 1980s (Denmark, Ireland, Italy, the Netherlands, Sweden, United Kingdom); meso‐level primary care reforms (Canada, Germany, Israel, Norway, Switzerland); and micro‐level reforms for cost containment (France).^37^ Enrolment was combined with initiatives for grouping physicians into larger practices,^28^, ^31^ to introduce multidisciplinary team‐based primary care,^30^, ^41^, ^42^, ^45^ and to specifically target people at high risk of poor health outcomes, including people with chronic health conditions or complex mental illness, and older people likely to have multiple health conditions.^30^, ^38^, ^40^, ^45^, ^46^ Enrolment was introduced in health systems with various models of primary care financing, including capitation, blended, and fee‐for‐service models.^31^, ^32^, ^37^, ^38^, ^40^, ^42^, ^45^, ^46^

Most enrolment schemes were voluntary, but the options varied markedly with regard to whether practices or patients were registered, where patients registered, which services were available to enrolled patients, and limitations or penalties for attending another practice. The only mandatory allocated scheme at the time registration was introduced was that in Italy.^37^

### Advantages and disadvantages of enrolment

The reported advantages of enrolment were related to clinical service delivery. Enrolment allowed better planning, the use of multidisciplinary teams, and greater efficiency and more income for practices.^25^, ^30^, ^45^

The multi‐jurisdictional study found that the effectiveness of patient registration for improving continuity of care and other health outcomes had been little investigated, particularly its effectiveness independent of other reforms.^37^ Country‐specific studies also found little or no effect of registration on continuity of care. In two countries where patients were already registered with practices, an additional requirement to register with an “accountable general practitioner” for people over 75 years of age (United Kingdom) or a “preferred doctor” (France) was introduced, suggesting that continuity of care might be better achieved at the general practitioner level;^24^, ^29^ neither study identified subsequent improvements in continuity of care.

The effect of enrolment accompanied by other reforms on emergency department use was mixed. Two studies in Ontario and one in Quebec found that the number of emergency department presentations and hospital admissions did not change;^25^, ^31^, ^34^ a second Quebec study found that emergency department use was reduced by 3%.^26^ The differences in the findings could be related to the enrolment process, associated reforms, or the study methodology.

In some studies, enrolment was found to be a barrier to primary care access and continuity of care, as indicated by waiting lists for registration;^27^, ^35^, ^41^, ^43^ some groups, often of marginalised people, not being registered;^32^, ^33^, ^36^, ^44^ and less continuity of care for people registered with a practice rather than a physician.^34^ Comparisons of jurisdictions with different models of enrolment and primary care found that some models suited some groups more than others;^14^, ^26^, ^28^ for example, enrolment of immigrants in Ontario was three times as high with capitation models as for family health teams.^33^ Enrolment of people with significant mental illness in Ontario was also lower than for the general population when the practice incentives to enrol people with complex needs were capped.^42^ (Box 3).

Box 3 **Advantages and disadvantages of patient enrolment in primary care reported by publications included in our scoping review**

**Table:** 

Advantages	Disadvantages
Benefits practice management in terms of workforce planning, efficiency^25^, ^30^, ^45^ Enables development and delivery of specific programs and interventions, such as screening, vaccinations, chronic disease management, follow‐up after hospitalisation, and after‐hours care^33^, ^34^, ^38^, ^40^, ^42^, ^45^, ^46^ Enables comparison of models of care^14^, ^26^, ^28^	Enrolment does not necessarily improve continuity of care (compared with seeing usual general practitioner), hospital/emergency department use, or other health outcomes^24^, ^25^, ^29^, ^31^, ^34^, ^37^, ^38^ Models that cap the number of patients often result in waiting lists for registration^27^, ^28^, ^35^, ^43^ and cherry‐picking of patients^27^, ^35^ Likelihood of enrolment^32^, ^33^, ^36^, ^42^, ^44^ and model of care preferences^33^ vary by socio‐demographic and health need characteristics Can reduce patient choice.^37^

### Enablers and barriers to enrolment

People are more likely to enrol with a general practitioner or practice when they perceive that it benefits them, regardless of the service funding model.^33^, ^39^ Their preference for continuity of care was indicated by the fact that their choice of “usual general practitioner” was not necessarily based on convenience or proximity.^23^, ^38^ Older people (for example, people aged 45 years or older^39^), women, people from higher socio‐economic areas, and people with chronic or multiple medical conditions were both more likely to enrol in voluntary registration schemes and to have usual general practitioners than younger people (for example, people aged 15–24 years^36^), men, and people from marginalised groups, including recent migrants and First Nations people.^27^, ^33^, ^36^, ^39^, ^44^

The multifaceted nature of primary care models made it unclear which enablers of and barriers to enrolment had the greatest impact. Practices were likely to register patients if encouraged by the payment model, regardless of the specific payment model.^8^, ^25^ Practices were discouraged from registering people if the model was complex or the capacity of the practice had been reached.^35^, ^41^ Further, funding models needed to adequately support practices to register people with complex health needs to ensure that the patients receive appropriate care^42^ and that practices do not register only people with fewer care needs^27^ (Box 4).

Box 4 **Enablers of and barriers to patient enrolment in primary care reported by publications included in our scoping review**

**Table:** 

Enablers	Barriers
Patients perceive benefit to enrolment, such as continuity of care^33^, ^39^ Patients have choice; ie, able to enrol with their usual general practitioner^23^, ^38^ Incentives to register; eg, better access to primary care, primary care at discounted rates or no cost, access to specialist care or subsidised specialist care, access to additional services (after‐hours care, post‐hospitalisation follow‐up)^23^, ^24^, ^33^, ^34^, ^36^, ^37^, ^38^, ^40^, ^42^, ^44^, ^45^, ^46^ Penalties for not registering; eg, higher cost of primary care services^24^ Adequate primary care services available^27^, ^35^, ^41^ Option to attend another practice if needed; eg, because of location or availability^23^, ^27^, ^35^, ^41^	Insufficient services, indicated by waiting lists^27^, ^35^, ^41^ Patients do not perceive or receive benefits from enrolment^38^ Patients cannot see their preferred general practitioner^34^ Patients prefer walk‐in models of care^33^

In summary, the characteristics of patient enrolment models in different countries differ greatly in both form and implementation. No specific model improved continuity of care while providing a mechanism for delivering other reforms.

## Discussion

The implications of the findings of our scoping review are relevant to the two objectives of the Australian patient enrolment reforms (MyMedicare) introduced in October 2023: to improve continuity of care, and to provide a platform for funding reform.^21^, ^47^

Contrary to expectations, we found little evidence that patient registration improves continuity of care. This finding may reflect strong affiliations with usual general practitioners prior to enrolment for people who would benefit most from continuity of care, including older people and those with chronic health conditions; further, both enrolment and research were focused on such people.^38^, ^42^, ^45^, ^46^ Improved continuity of care could be more noticeable among people who do not have usual general practitioners, but they were not investigated in the studies reviewed.

Enrolment was sometimes associated with reduced continuity of care, because of difficulty obtaining an appointment with a preferred general practitioner,^25^ care shifting from a usual general practitioner to another person in the practice,^34^ practices possibly focusing on people with less complex needs,^27^, ^35^, ^42^ or difficulty in enrolling with a practice,^27^, ^35^, ^41^ particularly for people from specific groups, such as recent migrants.^35^ As enrolment was often part of broader reform, change may be driven or limited by factors associated with other changes. If funding was inadequate or the reforms were complex, enrolment was associated with poorer outcomes for patients, general practitioners, and practices.^48^

We found that the nature of enrolment and associated reforms and rates of enrolment each varied according to the administrative mechanism, associated incentives, and the cultural and operational context. This included the degree of choice as to whether or where to enrol, the level of choice at the practice, and the incentives or disincentives associated with enrolment. For example, enrolment in some countries was associated with better access to services or financial incentives (lower out‐of‐pocket costs). Rates of enrolment were lower in schemes with weaker incentives. Enrolment mechanisms and rates should be further investigated to determine whether the same factors drive enrolment overall and for particular patient groups; those who are not currently experiencing continuity of care should be identified, as should those who could particularly benefit from enrolment. Enrolment is likely to benefit everyone with respect to relational and informational continuity, and may facilitate improved funding of primary care.

High enrolment rates are required to support primary care funding reforms; from the viewpoint of the health care system, the attachment of patients to usual general practitioners is insufficient, as they are not discouraged from visiting several practices.^19^ MyMedicare offers only limited incentives for practices, general practitioners, or patients that encourage enrolment or patients to use a single provider. Enrolment should be closely monitored to determine why practices and patients participate in enrolment, to ensure that the scheme facilitates continuity of care and further reforms. In addition to monitoring overall enrolment, the enrolment of specific groups who may experience barriers to health care access should be specifically monitored,^49^ including Aboriginal and Torres Strait Islander people, residents of rural and remote Australia, older Australians, people with mental illness or disability, people from culturally and linguistically diverse backgrounds, and LGBTIQ+ people.^20^

For MyMedicare to work, practices and patients need to see value in enrolment. This will be difficult to establish without more targeted evaluation of its benefits, not just for those who have usual general practitioners, but also for those who do not. The limited incentives offered by MyMedicare mean that it is an opportunity for studying the impact of patient registration on continuity of care when most people may not benefit from the incentives.

When reforms have multiple purposes, as with MyMedicare, their implementation requires consideration of how each aim will be achieved. Incentives are needed to encourage continuity of care for both practices and patients, supporting relational, informational, and management continuity of care. Incentives are also needed to encourage enrolment to enable the delivery of other reforms, including financial support for this behavioural change and its administration. The design of incentives should take potential unintended consequences into account and ensure equitable access.

### Limitations

First, our review of articles published during 2014–24 focused on more recent meso‐ and micro‐level primary care reforms.^37^ While recent reforms are likely to be more relevant to Australia, less recent macro‐level reforms, such as the introduction of the National Health Service in the United Kingdom, could also be relevant. Further, other schemes may not yet have been reported in the literature. Second, characteristics of enrolment schemes were reported differently in the included publications. Most were described as voluntary, but the choices and limitations for practices and patients differed substantially between and within jurisdictions. Third, continuity of care in primary care leads to better patient outcomes,^7^, ^11^, ^13^ and it is assumed that patient enrolment enables continuity of care and consequently better patient outcomes.^4^ However, as patients often prefer to see their usual general practitioners, enrolment may simply formalise an existing preference. The studies we included often involved patients likely to benefit most from continuity of care and therefore likely to already have preferred practitioners, such as people over 65 years of age or with chronic illnesses, so they did not have many additional benefits from enrolment. Fourth, people's preferences and behaviours can be deeply embedded and take time to change; patient behaviour and how patient enrolment can be most effective encouraged requires further investigation.

### Conclusions

Patient enrolment often has the dual purpose of improving continuity of care and supporting primary care service delivery and reforms. We found some evidence of enrolment improving efficiency in primary care delivery, but little that it improves continuity, quality, or the equity of primary care. This may reflect the fact that many people have preferred practitioners and thereby naturally select continuity of care. When different funding and enrolment models operate concurrently, unintended outcomes are possible, including people in marginalised groups or with complex care needs being less likely to enrol and use primary care. Further investigation of the diversity of patient enrolment schemes and their impact on both continuity of care and supporting primary care reform is needed, and the engagement of practitioners and patients with MyMedicare should be closely monitored, both overall and for specific groups of people.

## Open access

Open access publishing facilitated by University of New South Wales, as part of the Wiley – University of New South Wales agreement via the Council of Australian University Librarians.

## Competing interests

Luke Allen is a salaried general practitioner and received consultancy payments from the World Health Organization and World Bank for advisory work on primary care reform programs. Michael Wright chairs the Royal Australian College of General Practitioners funding and health system reform expert committee and is chief medical officer at Avant Mutual. Michael Kidd is former deputy chief medical officer with the Australian Department of Health and Aged Care, where he was involved in primary care reform developments.

## Supporting information


Supplementary methods and results

## References

[1] Organisation for Economic Cooperation and Development . Realising the potential of primary health care [OECD Health Policy Studies]. 30 May 2020. https://www.oecd‐ilibrary.org/social‐issues‐migration‐health/realising‐the‐potential‐of‐primary‐health‐care_a92adee4‐en (viewed Nov 2024).

[2] Azimzadeh S , Azami‐Aghdash S , Tabrizi JS , Gholipour K . Reforms and innovations in primary health care in different countries: scoping review. Prim Health Care Res Dev 2024; 25: e22. 38651337 10.1017/S1463423623000725 PMC11091477

[3] Australian Department of Health and Aged Care . Strengthening Medicare taskforce report. Dec 2022. https://www.health.gov.au/sites/default/files/2023‐02/strengthening‐medicare‐taskforce‐report_0.pdf (viewed Nov 2024).

[4] Bodenheimer T , Ghorob A , Willard‐Grace R , Grumbach K . The 10 building blocks of high‐performing primary care. Ann Fam Med 2014; 12: 166‐171. 24615313 10.1370/afm.1616 PMC3948764

[5] Wright M , Versteeg R . Introducing general practice enrolment in Australia: the devil is in the detail. Med J Aust 2021; 214: 400. https://www.mja.com.au/journal/2021/214/9/introducing‐general‐practice‐enrolment‐australia‐devil‐detail 33873246 10.5694/mja2.51027

[6] Pereira Gray DJ , Sidaway‐Lee K , White E , et al. Continuity of care with doctors: a matter of life and death? A systematic review of continuity of care and mortality. BMJ Open 2018; 8: e021161. 10.1136/bmjopen-2017-021161 PMC6042583 29959146

[7] Guthrie B , Saultz JW , Freeman GK , Haggerty JL . Continuity of care matters. BMJ 2008; 337: a867. 18687724 10.1136/bmj.a867

[8] Grumbach K , Olayiwola JN . Patient empanelment: the importance of understanding who is at home in the medical home. J Am Board Fam Med 2015; 28: 170‐172. 25748755 10.3122/jabfm.2015.02.150011

[9] Harris MF , Rhee J . Achieving continuity of care in general practice: the impact of patient enrolment on health outcomes. Med J Aust 2022; 216: 460‐461. https://www.mja.com.au/journal/2022/216/9/achieving‐continuity‐care‐general‐practice‐impact‐patient‐enrolment‐health 35443290 10.5694/mja2.51508

[10] Wright M , Mainous AG . Can continuity of care in primary care be sustained in the modern health system? Aust J Gen Pract 2018; 47: 667‐669. 31195767 10.31128/AJGP-06-18-4618

[11] Van Walraven C , Oake N , Jennings A , Forster AJ . The association between continuity of care and outcomes: a systematic and critical review. J Eval Clin Pract 2010; 16: 947‐956. 20553366 10.1111/j.1365-2753.2009.01235.x

[12] World Health Organization . Continuity and coordination of care: a practice brief to support implementation of the WHO Framework on integrated people‐centred health services. 7 Nov 2018. https://www.who.int/publications/i/item/9789241514033 (viewed July 2024).

[13] Haggerty JL , Reid RJ , Freeman GK , et al. Continuity of care: a multidisciplinary review. BMJ 2003; 327: 1219‐1221. 14630762 10.1136/bmj.327.7425.1219 PMC274066

[14] Kantarevic J , Kralj B , Weinkauf D . Enhanced fee‐for‐service model and physician productivity: evidence from family health groups in Ontario. J Health Econ 2011; 30: 99‐111. 21111500 10.1016/j.jhealeco.2010.10.005

[15] Santos F , Conti S , Wolters A . A novel method for identifying care home residents in England: a validation study. Int J Popul Data Sci 2020; 5: 1666. 34568584 10.23889/ijpds.v5i4.1666 PMC8441962

[16] Souty C , Turbelin C , Blanchon T , et al. Improving disease incidence estimates in primary care surveillance systems. Popul Health Metr 2014; 12: 19. 25435814 10.1186/s12963-014-0019-8 PMC4244096

[17] Vahabi M , Lofters A , Kumar M , Glazier RH . Breast cancer screening disparities among urban immigrants: a population‐based study in Ontario, Canada. BMC Public Health 2015; 15: 679. 26194189 10.1186/s12889-015-2050-5 PMC4508905

[18] Australian Bureau of Statistics . Patient experiences, 2022–23; here: table 5.3. 21 Nov 2023. https://www.abs.gov.au/statistics/health/health‐services/patient‐experiences/2022‐23#data‐downloads (viewed June 2024).

[19] Wright M , Hall J , van Gool K , Haas M . How common is multiple general practice attendance in Australia? Aust J Gen Pract 2018; 47: 289‐296. 29779298 10.31128/AJGP-11-17-4413

[20] Australian Department of Health . Future focused primary health care: Australia's primary health care 10 year plan 2022–2032. Mar 2022. https://www.health.gov.au/sites/default/files/documents/2022/03/australia‐s‐primary‐health‐care‐10‐year‐plan‐2022‐2032.pdf (viewed Nov 2024).

[21] Australian Department of Health and Aged Care . Information for MyMedicare patients. Updated 20 Aug 2024. https://www.health.gov.au/our‐work/mymedicare/patients (viewed Nov 2024).

[22] Tricco AC , Lillie E , Zarin W , et al. PRISMA extension for scoping reviews (PRISMA‐ScR): checklist and explanation. Ann Intern Med 2018; 169: 467‐473. 30178033 10.7326/M18-0850

[23] Carmody P , Whitford DL . Telephone survey of private patients’ views on continuity of care and registration with general practice in Ireland. BMC Fam Pract 2007; 8: 17. 17397546 10.1186/1471-2296-8-17 PMC1851962

[24] Dourgnon P , Guillaume S , Naiditch M , Ordonneau C . Introducing gate keeping in France: first assessment of the preferred doctor scheme reform [Issues in Health Economics, no. 124]. July 2007. https://www.irdes.fr/EspaceAnglais/Publications/IrdesPublications/QES124.pdf (viewed Nov 2024).

[25] Glazier RH , Klein‐Geltink J , Kopp A , Sibley LM . Capitation and enhanced fee‐for‐service models for primary care reform: a population‐based evaluation. CMAJ 2009; 180: E72–E81. 19468106 10.1503/cmaj.081316 PMC2683211

[26] Tiagi RA , Tiagi R , Chechulin Y . The effect of rostering with a patient enrolment model on emergency department utilization. Healthc Policy 2014; 9: e105‐e121. PMC4749888 24973487

[27] Breton M , Brousselle A , Boivin A , et al. Who gets a family physician through centralized waiting lists? BMC Fam Pract 2015; 16: 10. 25649074 10.1186/s12875-014-0220-7 PMC4328670

[28] Kiran T , Kopp A , Moineddin R , Glazier RH . Longitudinal evaluation of physician payment reform and team‐based care for chronic disease management and prevention. CMAJ 2015; 187: E494‐E502. 26391722 10.1503/cmaj.150579 PMC4646764

[29] Barker I , Lloyd T , Steventon A . Effect of a national requirement to introduce named accountable general practitioners for patients aged 75 or older in England: regression discontinuity analysis of general practice utilisation and continuity of care. BMJ Open 2016; 6: e011422. 10.1136/bmjopen-2016-011422 PMC5030554 27638492

[30] Christiansen E , Hampton MD , Sullivan M . Patient empanelment: a strategy to improve continuity and quality of patient care. J Am Assoc Nurse Pract 2016; 28: 423‐428. 26847151 10.1002/2327-6924.12341

[31] Strumpf E , Ammi M , Diop M , et al. The impact of team‐based primary care on health care services utilization and costs: Quebec's family medicine groups. J Health Econ 2017; 55: 76‐94. 28728807 10.1016/j.jhealeco.2017.06.009

[32] Burch P , Doran T , Kontopantelis E . Regional variation and predictors of over‐registration in English primary care in 2014: a spatial analysis. J Epidemiol Community Health 2018; 72: 532‐538. 29449351 10.1136/jech-2017-210176

[33] Batista R , Pottie KC , Dahrouge S , et al. Impact of health care reform on enrolment of immigrants in primary care in Ontario, Canada. Fam Pract 2019; 36: 445‐451. 30219848 10.1093/fampra/cmy082

[34] Singh J , Dahrouge S , Green ME . The impact of the adoption of a patient rostering model on primary care access and continuity of care in urban family practices in Ontario, Canada. BMC Fam Pract 2019; 20: 52. 30999868 10.1186/s12875-019-0942-7 PMC6474046

[35] Breton M , Smithman MA , Kreindler SA , et al. Designing centralized waiting lists for attachment to a primary care provider: considerations from a logic analysis. Eval Program Plann 2021; 89: 101962. 34127272 10.1016/j.evalprogplan.2021.101962

[36] Irurzun‐Lopez M , Jeffreys M , Cumming J . The enrolment gap: who is not enrolling with primary health organizations in Aotearoa New Zealand and what are the implications? An exploration of 2015–2019 administrative data. Int J Equity Health 2021; 20: 93. 33823865 10.1186/s12939-021-01423-4 PMC8025352

[37] Marchildon GP , Brammli‐Greenberg S , Dayan M , et al. Achieving higher performing primary care through patient registration: a review of twelve high‐income countries. Health Policy 2021; 125: 1507‐1516. 34531039 10.1016/j.healthpol.2021.09.001

[38] Bonney A , Russell G , Radford J , et al. Effectiveness of quality incentive payments in general practice (EQuIP‐GP) cluster randomized trial: impact on patient‐reported experience. Fam Pract 2022; 39: 373‐380. 35640205 10.1093/fampra/cmab157 PMC9155154

[39] Lavergne MR , King C , Peterson S , et al; QC‐BC Patient Enrolment Project Team. Patient characteristics associated with enrolment under voluntary programs implemented within fee‐for‐service systems in British Columbia and Quebec: a cross‐sectional study. CMAJ Open 2022; 10: E64‐E73. 10.9778/cmajo.20210043 PMC8812717 35105683

[40] Reed RL , Roeger L , Kwok YH , et al. A general practice intervention for people at risk of poor health outcomes: the Flinders QUEST cluster randomised controlled trial and economic evaluation. Med J Aust 2022; 216: 469‐475. https://www.mja.com.au/journal/2022/216/9/general‐practice‐intervention‐people‐risk‐poor‐health‐outcomes‐flinders‐quest 35388512 10.5694/mja2.51484 PMC9321612

[41] Smithman MA , Haggerty J , Gaboury I , Breton M . Improved access to and continuity of primary care after attachment to a family physician: longitudinal cohort study on centralized waiting lists for unattached patients in Quebec, Canada. BMC Primary Care. 2022; 23: 238. 36114464 10.1186/s12875-022-01850-4 PMC9482231

[42] Bayoumi I , Whitehead M , Li W , et al. Association of physician financial incentives with primary care enrolment of adults with serious mental illnesses in Ontario: a retrospective observational population‐based study. CMAJ Open 2023; 11: E1‐E12. 10.9778/cmajo.20210190 PMC9842098 36627127

[43] Nabieva K , McCutcheon T , Liddy C . Connecting unattached patients to comprehensive primary care: a rapid review. Prim Health Care Res Dev 2023; 24: e19. 36919838 10.1017/S1463423623000099 PMC10050950

[44] Pledger M , Mohan N , Silwal P , Irurzun‐Lopez M . The enrolment gap and the COVID‐19 pandemic: an exploration of routinely collected primary care enrolment data from 2016 to 2023 in Aotearoa New Zealand. J Prim Health Care 2023; 15: 316‐323. 38112703 10.1071/HC23128

[45] Javanparast S , Roeger L , Reed RL . General practice staff and patient experiences of a multicomponent intervention for people at high risk of poor health outcomes: a qualitative study. BMC Primary Care 2024; 25: 18. 38191349 10.1186/s12875-023-02256-6 PMC10775450

[46] Reed RL , Roeger L , Kaambwa B . Two‐year follow‐up of a clustered randomised controlled trial of a multicomponent general practice intervention for people at risk of poor health outcomes. BMC Health Serv Res 2024; 24: 488. 38641587 10.1186/s12913-024-10799-2 PMC11031969

[47] Australian Department of Health and Aged Care . Information for MyMedicare general practices and healthcare providers. Updated 30 Sept 2024. https://www.health.gov.au/our‐work/mymedicare/practices‐and‐providers (viewed Nov 2024).

[48] Pearse J , Mazevska D , McElduff P , et al. Evaluation of the Health Care Homes trial. Volume 1: summary report. 29 July 2022. https://www.health.gov.au/sites/default/files/documents/2022/08/evaluation‐of‐the‐health‐care‐homes‐trial‐final‐evaluation‐report‐2022.pdf (viewed Nov 2024).

[49] Bates S , Kayess R , Katz I . What can we learn from disability policy to advance our understanding of how to operationalise intersectionality in Australian policy frameworks? Australian Journal of Public Administration 2024; 10.1111/1467-8500.12648 (viewed Nov 2024).

