# Supplementary material for: The impact of patient enrolment in primary care on continuity and quality of care around the world, 2014–2024, and lessons for Australia: a scoping review
Source: Med J Aust. 2025 Apr 15;222(9):462–71. doi: 10.5694/mja2.52648 (PMC12088321; doi:10.5694/mja2.52648)
Supplement: Supplementary file 1 — Supplementary methods and results [file MJA2-222-462-s001.pdf]

The Medical Journal of Australia

Supporting Information

Supplementary methods and results

This appendix was part of the submitted manuscript and has been peer reviewed.
It is posted as supplied by the authors.

Appendix to: Bates SM, Lin J, Allen L, et al. The impact of patient enrolment in primary care on continuity and quality of care around the world, 2014–2024, and lessons for Australia: a scoping review. *Med J Aust* 2025; doi: 10.5694/mja2.52648.

Supplementary methods

1. MyMedicare

The new Australian scheme of patient enrolment, MyMedicare, includes eligibility requirements for both patients and practices. Practices must be accredited against the National General Practice Accreditation Scheme, although exemptions exist for Aboriginal Community Controlled Health Services and mobile outreach services accredited under the National Safety and Quality Primary and Community Healthcare Standards.[21] Patients can enrol in registered practices who have had at least two face-to-face visits with the practice in the previous 24 months, although exemptions exist for priority groups who may register on their first visit.

Patients registered under the MyMedicare scheme are provided greater continuity of care, longer telehealth consultations, and more opportunities to be bulk billed (for children under 16, pensioners and concession card holders); the reforms also provide more regular visits for people living in residential aged care homes, and connections to more appropriate care in general practice for people who present to hospitals frequently.[21] People who choose not to register can still access quality primary care services; further, registered patients may also attend other providers.[21]

The scheme is also supported by My Health Record, Australia's national electronic health record, which allows other health professionals to know who to talk to about an individual patient's regular care – although the system allows patients to choose to opt out of having their MyMedicare registration appear on their My Health Record.[21] Given the level of choice by practices and patients on whether to enrol, and the way in which they can engage with these reforms, this raised the question of what additional outcomes can be expected for patients from the new scheme.

For practices, My Medicare is expected to provide more information about regular patients, making it easier to tailor services to fit patient needs, and provide access to additional Medical Benefits Schedule (MBS) items, including longer telehealth calls, and a triple bulk billing incentive for longer MBS telehealth consultations for those under 16, pensioners and concession card holders.[48] Further, in 2024-2025, MyMedicare practices can access a General Practice in Aged Care Incentive (providing regular general practitioner visits to people in residential aged care), blended payments to support people with complex, chronic disease who frequently attend hospitals, and chronic disease management items to support care for patients with chronic and complex conditions in the community (also open to non-MyMedicare patients).[48]

Table 1. Screening process: inclusion criteria for studies to be including in the scoping review of published studies of patient enrolment in primary care

Types of studies
Research studies<br>Comparative research studies<br>Systematic reviews<br>Meta-analysis
Types of participants
Patients<br>General practice/primary health care facility
Types of interventions
Voluntary patient enrolment with general practitioners or primary care physicians<br>Compulsory patient enrolment with general practitioners or primary care physicians<br>Other models of patient enrolment
Types of outcome measures
Patient registration (voluntary, compulsory enrolment)*<br>Choice of where to register or empanelment (government or other)*<br>Register at practice or with particular doctor*<br>Patient–provider agreement, obligations of patients and providers*<br>Financial incentives for registration for patient or practitioner (capitation or bonuses)*<br>Other benefits of registration (additional services)<br>Rate of registration (percentage of population registered)<br>Access to primary care<br>Continuity of care<br>Quality of care<br>Preventive care<br>Health outcomes<br>Increased patient satisfaction<br>Decreased emergency department presentations and hospitalisations<br>Information continuity<br>Provider behaviour

* Study types included in an earlier multi-country review.[37]

Supplementary results

2. Summary of excluded articles

The table below provides a summary of articles determined not in scope after the full text review and the reason for their exclusion. As many provided useful contextual information, they are summarised in the tables below. Table 1 provides a list of articles determined not in scope listed in chronological order. he referencing is continuous from the main manuscript and includes papers included in the contextual background in the main manuscript.

Table 1. Summary of articles determined not in scope after full text review

Reference	Location	Overview	Reason excluded
Menec et al. (2001)[5]	Canada (Manitoba)	Examines scale of informal registration in Manitoba. Significant variation in informal registration (15-68%) and number of informally registered patients per physician (544-1378). Informal registration higher in rural practices (60%) than urban (38%).	Relates to informal registration rather than formal registration. Also out of date range.
Guthrie et al. (2008)[7]	N/A	Analysis about the importance of continuity of care and how a person's health care is connected over time. Current care cannot be isolated from past care or future care. Provides a breakdown of different types of continuity – informational, management and relationship – but recognises there is less agreement which dimensions are more important. They argue all dimensions are important.	About continuity of care – not voluntary enrolment. Also out of date range.
Kantarevic et al. (2011)[14]	Canada (Ontario)	Impact of payment model (Family Health Group – an enhanced fee for service model) on physician productivity. Shows increased physician productivity (6-10%) measured as number of services, patient visits, and distinct patients seen. Doctors also have lower referral rates and treat slightly more complex patients.	About physician productivity under different funding models. Enrolment is one part of the funding model. Also out of date range.
McRae et al. (2011)[51]	Australia	Examines what patients are 'de facto' affiliated with general practitioners, i.e. see a usual general practitioner in Australia. They found patients in poor or fair self-assessed health were relatively unlikely to see a usual general practitioner – therefore enrolment should target this group.	Need for enrolment. Also out of date range.
Glazier et al. (2012)[52]	Canada (Ontario)	Lessons from different patient enrolment models - blended capitation model and enhanced fee for service model. Administrative data analysis identifies practice characteristics, patterns of care, comprehensiveness of care, continuity of care, after hours care, visits to emergency departments and uptake of new patients, by payment model. Found variation in types of care provided – payment mechanisms had greater impact than enrolment.	Focus is on payment models, and practice characteristics of different funding models. Also out of date range.
Lewis and Longley (2012)[53]	United Kingdom (London)	Looks at patterns of registration in the United Kingdom - by geography and ethnicity. Authors highlight finds from National Health Service survey that people 'from black and ethnic minority groups and people living in more deprived areas ... want greater control of how and when they access primary care' (p1137).	Examines choice of practice registered with relative to geography and ethnicity. Also out of date range.
Souty et al. (2014)[16]	France (Paris)	Examines benefits of registration to improve disease incidence estimates in public health surveillance.	Use of registration data.
Sweetman and Buckley (2014)[54]	Canada (Ontario)	Overview of Ontario's primary care reform – summary of the history of reform and outcomes to date.	Contextual only.
Aysola et al. (2015)[55]	United States	Evaluation of patient centred medical homes, with purposive sampling of minority and non-minority groups with diabetes or hypertension to understand whether impact varied by type of model or patient race/ethnicity.	About change in outcomes by model/race rather than enrolment.
Bovet et al. (2015)[56]	Switzerland	Uses cardiovascular disease to highlight need for patient registration to facilitate screening and early intervention.	Reason to introduce registration rather than how to implement.

Reference	Location	Overview	Reason excluded
Ouellette-Kuntz (2015) [57]	Canada (Ontario)	Examines association of patient enrolment and health screening for people with intellectual and developmental disabilities. Finds seeing a general practitioner in a patient enrolment model means more likely to have been screened for bowel cancer.	Association between screening and enrolment, rather than enablers/barriers to enrolment.
Rudoler et al. (2015a) [58]	Canada (Ontario)	Analyses risk selection in capitation based models of primary care to understand relationship between physician payments, risk selection and health care costs. They find a relationship between capitation payments and low cost payments.	About payment mechanisms rather than enrolment
Rudoler et al. (2015b) [59]	Canada (Ontario)	Examines physicians' self-selection into different payment models grouped by fee for service, enhanced fee for service, and blended capitation. Physicians more likely to self-select into schemes based on existing characteristics. Patients with more complex patients less likely to switch to capitation based models where effort was not rewarded.	About variations in demographics by funding model
Vahabi et al. (2015)[17]	Canada (Ontario)	Disproportionate number of women who are recent immigrants die of breast cancer. This study examines screening rates and why they differ for this population. 64% of cohort were screened. Lower screening rates were associated with living in low SES neighbourhoods, having a male general practitioner, having an internationally trained general practitioner, and not being enrolled in one of the primary health care models. Time in Canada, age, comorbidities, higher health service use were also a factor. Those not enrolled with a practice 22% less likely to be screened. Recommend increasing this groups access to enrolment models and female general practitioners.	Association between breast cancer screening rates and several factors – one of which was primary care model and registration.
Marchildon and Hutchison (2016)[60]	Canada (Ontario)	Narrative of earlier reforms and proposals made in 2015. Reforms included changes to remuneration, performance incentives, and bonuses, and supported by increase from 7.5% to 8.1% of total health care expenditure.<br>Reforms did not achieve improvements in access and quality - subsequently government now seeking to contain costs. Capitation and team based models disproportionately attracted physicians serving affluent, healthy, low-cost populations - raising equity concerns. Reforms took a long time to have any effect - now more people surveyed were likely to have a regular family doctor (91% compared to national average of 84%) and rated quality of services as excellent or very good.	Contextual narrative of the reforms and the proposals made in 2015
McLeod et al. (2016)[61]	Canada (Ontario)	Descriptive study of different primary care models including patient enrolment, financing, from the perspective of physicians rather than patients. Funding models are associated with different characteristics of physicians. Patients in group based models often don't see the general practitioner group they are rostered to.	Highlights variations in general practitioner demographics by funding model, number of patients by funding model, and workload
Satre et al. (2016) [62]	United States (California)	Looks at impact of enrolment in a program and change to benefit structures on coordination of health care for HIV-positive patients.	Enrolment but for specialist care
Vahabi et al. (2016)[63]	Canada (Ontario)	Examines the association between breast screening rates for women from Muslim majority countries and several other factors – one of which is registration.	Association between breast cancer screening rates and several factors – one of which was primary care model and registration.
Riordan et al. (2017)[64]	Ireland	Using data from 1998, 2002, 2008 and 2015, examines documentation of care processes and outcomes for diabetes patients registered at participating practices. Results show improvements in the documentation of care over time and patient outcomes.	Insufficient detail – conference abstract and enablers/barriers to enrolment unknown.
Vahabi et al. (2017)[65]	Canada (Ontario)	Examines the association between breast screening rates for women from Muslim majority countries and several other factors – one of which is registration.	Association between breast cancer screening rates and several factors – one of which was primary care model and registration.

Reference	Location	Overview	Reason excluded
Bearden et al. (2019)[66]	Multiple	Establishes standard concepts for empanelment and why and how empanelment is used. Develops guidance for implementation of empanelment in low and middle income countries using a literature review and a multi-country collaborative.	About how to empanel patients - not voluntary enrolment.
Laberge and Gaudreault (2019)[67]	Canada (Quebec)	Talks about reasons to promote access to family medicine group and reason for reforms. Find increase (from 68% to 81%) in population registering with primary physician (short of 85% target). Continuity of care increased from 68% to 84%. However, model made general practice less attractive to medical students (media releases on physician income highlighted disparity between general practice and specialists). Also general practitioners threatened to retire or relocate, as they were not prepared to meet quotas.	Descriptive paper about mechanisms for reform.
Thomas et al. (2019)[68]	Australia	Examines what Australian general practitioners anticipate from the Health Care Homes reforms trial in terms of affecting whole of person care. General practitioners queried the design and the different funding models for acute and chronic care of the same patient. Some involved in the trial also reported practical issues and potential gaming.	About health care homes model not enrolment
Whitehead et al. (2019)[69]	New Zealand	Looks at proximity of registration and spatial equity. Specifically, factors contributing to patients bypassing most local general practitioner. 68% patients bypassed service closest to home. Varied by rural and urban areas. Also associated with ethnicity, age, SES, sex, distance to clinic, after hours availability, Māori service provider status, general practitioner/Nurse FTE, and clinic fees. People in rural areas living more than 20km from closest general practitioner service had high rates of general practitioner bypass.	Proximity of practice registered with – not the registration mechanism.
Breton et al. (2020)[70]	Multiple	To understand how to design and implement centralised waiting lists. Review of 21 articles.	Implementation study of centralised waiting lists
Fiset-Laniel et al. (2020)[71]	Canada (Quebec)	Looks at whether registration with 'interprofessional' family medicine groups impact on rates of screening (bowel cancer, breast cancer, and osteoporosis) for patients. Found no evidence that attending a family medicine group affected screening rates. Patients attending other general practitioners (not enrolled) had similar rates.	Focus is on impact of different models of primary care on screening rates, rather than enrolment
Marchildon et al.(2020)[72]	Comparison of registration practices in ten jurisdictions	Describes the intended study, later reported in Marchildon et al. 2021.	Scope of study reported in Marchildon et al. 2021.
Santos et al. (2020)[15]	United Kingdom	Use of patient registration data to identify whether patients are living in aged care.	Use of registration data.
Whitehead et al. (2020)[73]	New Zealand	Considers equity of access to primary care by comparing enrolment data with location of payment.	Proximity of practice registered with – not the registration mechanism.
Ly et al. (2021)[74]	Canada (Ontario)	Examines emergency departments use and whether different primary care models are associated with non-urgent emergency departments use in Hamilton (neighbourhood with relatively high marginalisation) relative to other populations. Examines whether there is value in conducting region specific assessments of primary care models. Models do work differently for different populations.	Examines impact of different primary care models on non-urgent emergency departments presentations.
Aoki et al. (2022a)[75]	Japan	Examines access to 'usual source of care' during the COVID-19 pandemic. Uses the patient experience survey (Japanese version of the Primary Care Assessment Tool Short-Form – JPCAT-SF). Almost one-fifth had restricted access to usual general practitioner for COVID-19 consultation during pandemic.	Having a usual general practitioner rather than enrolment.

Reference	Location	Overview	Reason excluded
Aoki et al. (2022b)[76]	Japan	Examines the relationship between having a usual primary care provider and preventive care measures during the COVID-19 pandemic. Having a usual source of primary care was positively associated with all preventive care composites - even during the COVID-19 pandemic. 57.5% of people surveyed had a usual general practitioner. This group was more likely to older, female, unemployed, and have more chronic health conditions.	Having a usual general practitioner rather than enrolment.
Harris and Rhee (2022)[9]	Australia	Editorial rather than research. Authors refer to studies that do not find improvements through registration - but recognise that many people already have a preferred general practitioner, so baseline is already high.	Editorial only
O'Loughlin et al. (2022)[77]	Australia (Queensland)	Qualitative study of patients with chronic conditions to inform medical home care model. People with chronic disease tended to stay with one general practitioner as they had their records - and prefer to see that general practitioner rather than other general practitioners or other services in the practice. Ideally this was someone who understood their needs, but some were unsatisfied but did not change as it would be hard to transition given their complex history. Participants preferred to see their regular general practitioner rather than others (and some would go to emergency departments if general practitioner unavailable)	Examines informal registration and why patients with chronic conditions are likely to have a usual general practitioner.
Snyder et al. (2022)[78]	United States (Ohio)	Introducing patient empanelment and seeing impact on well childcare visits and timely access to doctors within a university teaching setting (170 residents train, providing care in 5 of 12 offices). Trial empanelled 90% patients to providers with sufficient availability to see them in their office. Then aimed to deliver continuity of an intervention. Introduced better structure and scheduling of clinicians to what was a highly variable service. Increase in 'show rates' (76.9% compared to 71.4%) for empanelled patients. Also decrease in emergency departments visits (20.5/1000 to 16.3 visits per 1000) - 20.5% decline. Increase in checks completed for empanelled group. All these points linked to attendance.	Examines empanelment in paediatric care (not primary care).
Sourial et al. (2022)[79]	Canada (Ontario)	Compares emergency departments use of patients with dementia attending a single general practitioner and those attending an interprofessional primary care team	Patient outcomes via different models of primary care.
True et al. (2022) [80]	Australia	Lessons from implementation of health care homes - specifically implementing change. This is part of a broader evaluation of the education and training provided for health care homes implementation.	About facilitating the implementation of the complex model – not enrolment
Aggarwal et al. (2023)[81]	Canada	Compares 13 Canadian jurisdictions towards high performing primary care. Highlights similarities and differences between jurisdictions, informed by interviews and desk top review.	About broader reforms – not enrolment.
Delpech et al. (2023)[82]	France (Paris)	Observational survey looking at presence of general practitioners, whether they were accepting new patients for office visits, and whether accepting new patients for home visits.	General practitioner supply issues, associated with practices capacity and willingness to take on new registrations – rather than how to implement registration
Moran et al. (2023)[83]	Multiple	Explores efficiency of primary care systems in European countries and the associations between efficiency and health system characteristics – the focus is on diabetes care.	About efficiency of primary care – not enrolment
Thekkur et al. (2023)[84]	Sri Lanka	Examines registration of individuals with primary medical care institutions (includes primary medical care units, divisional hospitals and some healthy lifestyle centres that provide preventive non-communicable disease care) as a precursor to empanelment (as part of a strengthening primary care project in Sri Lanka)	About broader health care reform and registration for health number and health records – precursor to empanelment
Tran et al. (2024)[85]	Australia	Examined the impact of Health Care Homes trial on quality of care and patient outcomes for trial participants (people with chronic health conditions) in Australia. While the trial provided greater access to care, there were no changes in health outcomes.	About health outcomes – not enrolment

References

7. Guthrie B, Saultz JW, Freeman GK, Haggerty JL. Continuity of care matters. BMJ Clinical Research Edition. 2008;337:a867.

9. Harris MF, Rhee J. Achieving continuity of care in general practice: the impact of patient enrolment on health outcomes. Med J Aust. 2022;216:460–1.

14. Kantarevic J, Kralj B, Weinkauf D. Enhanced fee-for-service model and physician productivity: evidence from family health groups in Ontario. J Health Econ. 2011;30:99–111.

15. Santos F, Conti S, Wolters A. A novel method for identifying care home residents in England: a validation study. Int J Popul Data Sci. 2020;5:09.

16. Souty C, Turbelin C, Blanchon T, Hanslik T, Le Strat Y, Boëlle PY. Improving disease incidence estimates in primary care surveillance systems. Popul Health Metr. 2014;12:19.

17. Vahabi M, Lofters A, Kumar M, Glazier RH. Breast cancer screening disparities among urban immigrants: a population-based study in Ontario, Canada. BMC Public Health. 2015;15:679.

21. Australian Government Department of Health and Aged Care. Information for MyMedicare patients. Webpage https://www.health.gov.au/our-work/mymedicare/patients#benefits. 2024 (accessed 8 November 2024).

22. Tricco AC, Lillie E, Zarin W, O'Brien KK, Colquhoun H, Levac D, et al. PRISMA extension for scoping reviews (PRISMA-ScR): checklist and explanation. Ann Intern Med. 2018;169:467–73.

37 Marchildon GP, Brammli-Greenberg S, Dayan M, et al. Achieving higher performing primary care through patient registration: a review of twelve high-income countries. Health Policy 2021; 125: 1507-1516.

48. Australian Government Department of Health and Aged Care. Information for MyMedicare general practices and healthcare providers. Webpage https://www.health.gov.au/our-work/mymedicare/practices-and-providers#benefits. 2024 (accessed 8 November 2024).

50. Menec V, Black C, Roos N, Bogdanovic B. What is the potential for formal patient registration in Canadian primary care? The scale of "informal registration" in Manitoba. J Health Serv Res Policy. 2001;6:202–6.

51. McRae I, Yen L, Gillespie J, Douglas K. Patient affiliation with GPs in Australia: who is and who is not and does it matter? Health Policy (New York). 2011;103:16–23.

52. Glazier RH, Kopp A, Schultz SE, Kiran T, Henry DA. All the right intentions but few of the desired results: lessons on access to primary care from Ontario's patient enrolment models. Healthc Q. 2012;15:17–21.

53. Lewis DJ, Longley PA. Patterns of patient registration with primary health care in the UK National Health Service. Ann Assoc Am Geogr. 2012;102:1135–45.

54. Sweetman A, Buckley G. Ontario's experiment with primary care reform. SPP Research Papers, The School of Public Policy, University of Calgary. 2014;7:1-37.

55. Aysola J, Werner RM, Keddem S, SoRelle R, Shea JA. Asking the patient about patient-centered medical homes: a qualitative analysis. J Gen Intern Med. 2015;30:1461–7.

56. Bovet P, Chiolero A, Paccaud F, Banatvala N. Screening for cardiovascular disease risk and subsequent management in low and middle income countries: challenges and opportunities. Public Health Rev. 2015;36:13.

57. Ouellette-Kuntz H, Coo H, Cobigo V, Wilton AS. Uptake of colorectal cancer screening among Ontarians with intellectual and developmental disabilities. PLoS One. 2015;10:e0118023.

58. Rudoler D, Laporte A, Barnsley J, Glazier RH, Deber RB. Paying for primary care: a cross-sectional analysis of cost and morbidity distributions across primary care payment models in Ontario Canada. Soc Sci Med. 2015;124:18–28.

59. Rudoler D, Deber R, Barnsley J, Glazier RH, Dass AR, Laporte A. Paying for primary care: the factors associated with physician self-selection into payment models. Health Econ. 2015;24:1229–42.

60. Marchildon GP, Hutchison B. Primary care in Ontario, Canada: new proposals after 15 years of reform. Health Policy (New York). 2016;120:732–8.

61. McLeod L, Buckley G, Sweetman A. Ontario primary care models: a descriptive study. CMAJ Open. 2016;4:E679–88.

62. Satre DD, Altschuler A, Parthasarathy S, Silverberg MJ, Volberding P, Campbell CI. Implementation and operational research: Affordable Care Act implementation in a California health care system leads to growth in HIV-positive patient enrollment and changes in patient characteristics. JAIDS. 2016;73:e76–82.

63. Vahabi M, Lofters A, Kumar M, Glazier RH. Breast cancer screening disparities among immigrant women by world region of origin: a population-based study in Ontario, Canada. Cancer Med. 2016;5:1670–86.

64. Riordan F, McHugh S, Harkins V, Kearney P. Trends in the quality of structured diabetes care in primary care. Int J Integr Care. 2017;17:172.

65. Vahabi M, Lofters A, Kim E, Wong JPH, Ellison L, Graves E, et al. Breast cancer screening utilization among women from Muslim majority countries in Ontario, Canada. Prev Med (Baltim). 2017;105:176–83.

66. Bearden T, Ratcliffe HL, Sugarman JR, Bitton A, Anaman LA, Buckle G, et al. Empanelment: a foundational component of primary health care. Gates Open Res. 2019;3:1–10.

67. Laberge M, Gaudreault M. Promoting access to family medicine in Québec, Canada: analysis of Bill 20, enacted in November 2015. Health Policy. 2019;123:901–5.

68. Thomas H, Best M, Mitchell G. Health care homes and whole-person care: a qualitative study of general practitioners' views. Aust J Gen Pract. 2019;48:867–74.

69. Whitehead J, Pearson AL, Lawrenson R, Atatoa-Carr P. Spatial equity and realised access to healthcare: a geospatial analysis of general practitioner enrolments in Waikato, New Zealand. Rural Remote Health. 2019;19:5349.

70. Breton M, Smithman MA, Sasseville M, Kreindler SA, Sutherland JM, Beauséjour M, et al. How the design and implementation of centralized waiting lists influence their use and effect on access to healthcare: a realist review. Health Policy. 2020;124:787–95.

71. Fiset-Laniel J, Diop M, Provost S, Strumpf EC. The impact of team-based primary care on guideline-recommended disease screening. Am J Prev Med. 2020;58:407–17.

72. Marchildon GP, Allin S, Quentin W. Strengthening primary care through patient registration: a review of 10 countries. Eur J Public Health. 2020;5:v491.

73. Whitehead J, Pearson AL, Lawrenson R, Atatoa-Carr P. Defining general practitioner and population catchments for spatial equity studies using patient enrolment data in Waikato, New Zealand. Appl Geogr. 2020;115:102137.

74. Ly O, Price D, Saskin R, Howard M. Low-acuity emergency department use among patients in different primary care models in Hamilton and Ontario. Healthc Manage Forum. 2021;34:234–9.

75. Aoki T, Fujinuma Y, Matsushima M. Patient experience of residents with restricted primary care access during the COVID-19 pandemic. Fam Med Community Health. 2022;10:e001667.

76. Aoki T, Fujinuma Y, Matsushima M. Usual source of primary care and preventive care measures in the COVID-19 pandemic: a nationwide cross-sectional study in Japan. BMJ Open. 2022;12:e057418.

77. O'Loughlin M, West C, Mills J. Medical homes and chronic care: consumer lessons for regional Australia. Aust J Prim Health. 2022;28:97–103.

78. Snyder DA, Schuller J, Ameen Z, Toth C, Kemper AR. Improving patient-provider continuity in a large urban academic primary care network. Acad Pediatr. 2022;22:305–12.

79. Sourial N, Schuster T, Bronskill SE, Godard-Sebillotte C, Etches J, Vedel I. Interprofessional primary care and acute care hospital use by people with dementia: a population-based study. Ann Fam Med. 2022;20:512–8.

80. True A, Janamian T, Dawda P, Johnson T, Smith G. Lessons from the implementation of the health care homes program. Med J Aust. 2022;216:S19–21.

81. Aggarwal M, Hutchison B, Abdelhalim R, Baker GR. Building high-performing primary care systems: after a decade of policy change, is Canada "walking the talk?" Milbank Q. 2023;101:1139–90.

82. Delpech R, Neindre C Le, Panjo H, Rigal L. Presence of primary care physicians and patients' ability to register: a simulated-patient survey in the Paris region. Ann Fam Med. 2023;21:341–3.

83. Moran V, Suhrcke M, Nolte E. Exploring the association between primary care efficiency and health system characteristics across European countries: a two-stage data envelopment analysis. BMC Health Serv Res. 2023;23:1348.

84. Thekkur P, Nair D, Fernando M, Kumar AMV, Satyanarayana S, Chandraratne N, et al. Empanelment of the population to the primary medical care institution of Sri Lanka: a mixed-methods study on outcomes and challenges. Healthcare. 2023;11:575.

85. Tran DT, Falster MO, Pearse J, Mazevska D, McElduff P, Pearson S, et al. The Australian Health Care Homes trial: quality of care and patient outcomes. A propensity score-matched cohort study. Med J Aust. 2024;220:372–8.

PRISMA-ScR checklist. Preferred Reporting Items for Systematic reviews and Meta-Analyses extension for Scoping Reviews (PRISMA-ScR) Checklist.[22]

Note: the page numbers refer to the submitted manuscript, not the published article or its supplementary information file.

SECTION	ITEM	PRISMA-ScR CHECKLIST ITEM	REPORTED ON PAGE #
	**TITLE**		
Title	1	Identify the report as a scoping review.	1
	**ABSTRACT**		
Structured summary	2	Provide a structured summary that includes (as applicable): background, objectives, eligibility criteria, sources of evidence, charting methods, results, and conclusions that relate to the review questions and objectives.	1
	**INTRODUCTION**		
Rationale	3	Describe the rationale for the review in the context of what is already known. Explain why the review questions/objectives lend themselves to a scoping review approach.	2-3
Objectives	4	Provide an explicit statement of the questions and objectives being addressed with reference to their key elements (e.g., population or participants, concepts, and context) or other relevant key elements used to conceptualize the review questions and/or objectives.	3
	**METHODS**		
Protocol and registration	5	Indicate whether a review protocol exists; state if and where it can be accessed (e.g., a Web address); and if available, provide registration information, including the registration number.	N/A Information is presented in methods and supplementary material
Eligibility criteria	6	Specify characteristics of the sources of evidence used as eligibility criteria (e.g., years considered, language, and publication status), and provide a rationale.	5
Information sources*	7	Describe all information sources in the search (e.g., databases with dates of coverage and contact with authors to identify additional sources), as well as the date the most recent search was executed.	4
Search	8	Present the full electronic search strategy for at least 1 database, including any limits used, such that it could be repeated.	4
Selection of sources of evidence	9	State the process for selecting sources of evidence (i.e., screening and eligibility) included in the scoping review.	5 and Box 1
Data charting process	10	Describe the methods of charting data from the included sources of evidence (e.g., calibrated forms or forms that have been tested by the team before their use, and whether data charting was done independently or in duplicate) and any processes for obtaining and confirming data from investigators.	N/A
Data items	11	List and define all variables for which data were sought and any assumptions and simplifications made.	5
Critical appraisal of individual sources of evidence	12	If done, provide a rationale for conducting a critical appraisal of included sources of evidence; describe the methods used and how this information was used in any data synthesis (if appropriate).	N/A
Synthesis of results	13	Describe the methods of handling and summarizing the data that were charted.	5
	**RESULTS**		
Selection of sources of evidence	14	Give numbers of sources of evidence screened, assessed for eligibility, and included in the review, with reasons for exclusions at each stage, ideally using a flow diagram.	6 and Figure 1
Characteristics of sources of evidence	15	For each source of evidence, present characteristics for which data were charted and provide the citations.	Table 1

SECTION	ITEM	PRISMA-ScR CHECKLIST ITEM	REPORTED ON PAGE #
Critical appraisal within sources of evidence	16	If done, present data on critical appraisal of included sources of evidence (see item 12).	N/A
Results of individual sources of evidence	17	For each included source of evidence, present the relevant data that were charted that relate to the review questions and objectives.	Table 1
Synthesis of results	18	Summarize and/or present the charting results as they relate to the review questions and objectives.	5-8
	**DISCUSSION**		
Summary of evidence	19	Summarize the main results (including an overview of concepts, themes, and types of evidence available), link to the review questions and objectives, and consider the relevance to key groups.	8-10
Limitations	20	Discuss the limitations of the scoping review process.	10
Conclusions	21	Provide a general interpretation of the results with respect to the review questions and objectives, as well as potential implications and/or next steps.	11
	**FUNDING**		
Funding	22	Describe sources of funding for the included sources of evidence, as well as sources of funding for the scoping review. Describe the role of the funders of the scoping review.	Title page
